# Lepra en la isla colombiana de Providencia

**DOI:** 10.7705/biomedica.4974

**Published:** 2020-08-20

**Authors:** Jairo Fuentes, Juliana Jiménez, Gustavo Urueta, Santiago Fadul, Esperanza Meléndez, Martha Inírida Guerrero, Gerzaín Rodríguez

**Affiliations:** 1 Servicio de Dermatología, Hospital Universidad del Norte, Departamento Administrativo de Salud del Distrito de Barranquilla, Colombia Hospital Universidad del Norte Departamento Administrativo de Salud Barranquilla Colombia; 2 Servicio de Dermatología, Hospital Militar Central, Bogotá, D.C., Colombia Hospital Militar Central BogotáD.C Colombia; 3 Secretaría Departamental de Salud, Archipiélago de San Andrés, Providencia y Santa Catalina, Colombia Secretaría Departamental de Salud Colombia; 4 Grupo de Epidemiología Aplicada, Instituto Nacional de Salud, Bogotá, D.C., Colombia Grupo de Epidemiología Aplicada Instituto Nacional de Salud BogotáD.C Colombia; 5 Servicio de Dermatología, Hospital Universidad del Norte, Departamento Administrativo de Salud del Atlántico, Barranquilla, Colombia Hospital Universidad del Norte Departamento Administrativo de Salud del Atlántico Barranquilla Colombia; 6 Oficina de Docencia e Investigación, Hospital Universitario Centro Dermatológico Federico Lleras Acosta, Bogotá, D.C., Colombia Hospital Universitario Centro Dermatológico Federico Lleras Acosta BogotáD.C Colombia; 7 Facultad de Medicina, Universidad de la Sabana, Chía, Colombia Universidad de la Sabana Facultad de Medicina Universidad de la Sabana Chía Colombia

**Keywords:** lepra multibacilar, lepra/transmisión, transmisión de enfermedad infecciosa, reacción en cadena de la polimerasa, Leprosy, multibacillary, leprosy/transmission, disease transmission, infectious, polymerase chain reaction

## Abstract

San Andrés y Providencia son islas colombianas en el mar de las Antillas. San Andrés tiene 68.283 habitantes y allí se han registrado casos de lepra en inmigrantes provenientes del interior colombiano. Providencia tiene 5.037 habitantes e, históricamente, los programas de salud no tenían registros de la enfermedad; no obstante, en el 2009 se confirmaron dos casos de lepra multibacilar histioide y, posteriormente, otros dos, lo cual representa una prevalencia de 8 casos por 10.000 habitantes y la convierte en un sitio hiperendémico para lepra. Inicialmente, se diagnosticó lepra histioide en una niña de 14 años y, durante su estudio, se encontró la misma forma clínica de la enfermedad en su padre. Recientemente, se detectó lepra multibacilar en otro miembro de la misma familia y, lepra indeterminada, en una niña de otro núcleo familiar.

El objetivo de este trabajo fue presentar estos casos clínicos ante la comunidad científica y los entes de salud pública, y llamar la atención de las autoridades de salud sobre la necesidad de establecer programas de vigilancia epidemiológica continua en la isla, incorporando las nuevas herramientas disponibles en el Programa de Control de la Lepra.

## Abstract (en)

San Andrés and Providencia are Colombian islands in the Caribbean Sea. San Andrés has 68,283 inhabitants and has registered cases of leprosy in immigrants from continental Colombia. Providencia has 5,037 inhabitants and historically health programs did not have records of the disease, but in 2009 two cases of multibacillary histoid leprosy were confirmed and, subsequently, another two, which represents a prevalence of 8 cases per 10,000 inhabitants and places the island as a hyperendemic site for leprosy. Initially, a 14-year-old girl with histoid leprosy was diagnosed and, exploring this case, her father was diagnosed with the same clinical form of leprosy. Recently, a new intrafamilial patient with multibacillary leprosy and an extrafamilial case of a girl with undetermined leprosy were detected.

The objective of this study was to present to the scientific community and the public health officers these clinical cases and to draw the attention of the sanitary authorities on the necessity of establishing continuous programs of leprosy epidemiological surveillance on the island using the new tools available in the *Programa de Control de la Lepra* (Leprosy Control Program).

La lepra está presente en todo el territorio colombiano, con mayor prevalencia en los departamentos de Norte de Santander, Santander, Huila, Cesar y Vichada ^1^. Anualmente, en Colombia se detectan alrededor de 450 nuevos casos, 75 % de los cuales son multibacilares ^1^.

En una comunidad, la lepra se extiende de manera semejante a las ondas que produce una piedra arrojada al agua estancada y tranquila ^2^. Los contactos intradomiciliarios del caso índice -aquellos que comparten techo y cocina- tienen una probabilidad diez veces mayor de contraer la lepra, comparados con la población general, así como una posibilidad 4 a 5 veces mayor de adquirirla que sus vecinos de cuadra y de barrio (peridomiciliarios) ^3^. Estos riesgos se incrementan cuando el caso índice es multibacilar y existe consanguinidad entre los contactos intradomiciliarios ^3^.

Los enfermos de lepra pueden albergar el bacilo en la capa córnea de su piel o en su nariz, desde donde continuamente lo eliminan, amplificando así su dispersión en la comunidad ^4^^,^^5^. El nuevo huésped lo puede adquirir por vía nasal o por la piel ^4^^,^^5^.

Las personas que se infectan con el bacilo pueden controlarlo mediante su sistema inmunitario en un plazo cercano a un año o, eventualmente, pueden desarrollar la lepra después de un periodo de incubación que generalmente varía entre 2 y 10 años ^5^. Cerca del 90 % de las personas infectadas son capaces de contrarrestar el bacilo y no presentar la enfermedad ^6^. Sin embargo, puede haber variaciones importantes, como epidemias o contagio, en personas de regiones previamente no expuestas al bacilo de la lepra ^7^^,^^8^.

Las personas con infección subclínica de lepra que alojen el bacilo en su nariz pueden esparcirlo en la comunidad ^5^. La infección subclínica se puede demostrar mediante métodos moleculares que detectan el ADN del bacilo de Hansen en el moco nasal ^9^ y por la presencia de anticuerpos IgM contra el glucolípido fenólico de la pared celular del bacilo (GLP-1) circulantes en la sangre del individuo infectado ^9^^,^^10^. En 80 a 90 % de los casos, la lepra comienza como una mancha hipocrómica e hipoestésica, etapa en que se la denomina ‘lepra indeterminada’ y puede diagnosticarse clínicamente ^11^.

En el 2009, se confirmaron dos casos de lepra multibacilar histioide en la isla de Providencia, donde los programas de salud no tenían registros de la enfermedad ^1^. El primero fue en una adolescente de 14 años y, posteriormente, se encontró la misma forma clínica de la enfermedad en su padre.

El objetivo de este trabajo fue presentar estos casos clínicos ante la comunidad científica y los entes de salud pública, informar sobre un nuevo caso intrafamiliar, y documentar un nuevo caso de lepra indeterminada en otro núcleo familiar cuyo seguimiento no ha sido posible. Llamamos la atención de las autoridades de salud sobre la necesidad de establecer programas de vigilancia epidemiológica continua de la lepra en la isla, usando herramientas clínicas, de laboratorio y de biología molecular.

## Consideraciones éticas

Esta publicación recibió el aval de los pacientes involucrados. Los datos de los pacientes se manejaron en forma anónima.

## Casos clínicos

***Caso 1*.** Se trata de una adolescente de 14 años de edad, estudiante, natural y procedente de Providencia. Seis años atrás había comenzado a presentar pápulas y nódulos cutáneos asintomáticos, los cuales fueron aumentando en número.

**Figura 1:**
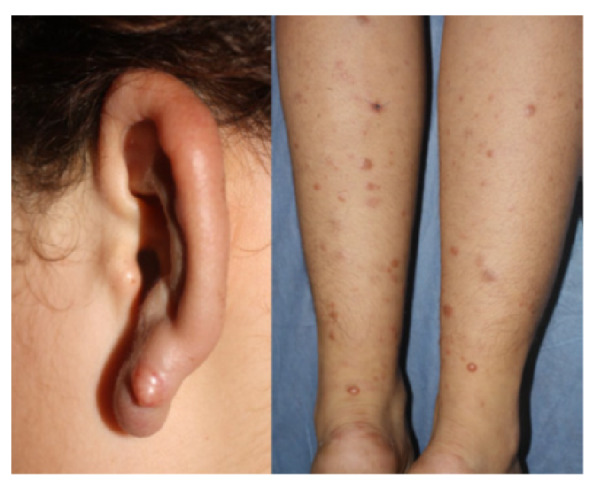
A. Paciente de 14 años con engrosamiento auricular y nódulo en la porción inferior de la oreja. B. Pápulas y nódulos en ambas piernas

En el examen físico se detectaron numerosos nódulos amarillentos o del color de la piel, en las orejas, los brazos, los muslos y las piernas (figura 1). No se encontraron máculas pigmentadas en los pliegues o en otras zonas, ni se detectó engrosamiento neural o alteración clara de la sensibilidad. La mucosa nasal era normal y la paciente no presentaba ningún grado de discapacidad.

**Figura 2:**
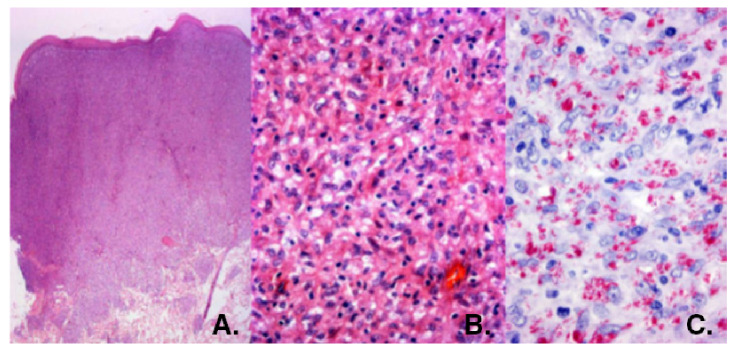
A. Biopsia de una pápula de la pierna. Infiltración dérmica difusa cubierta por epidermis atrófica. Hematoxilina y eosina, 2,5X. B. El infiltrado consta de macrófagos, algunos vacuolados, con pocos linfocitos y algunos plasmocitos. Hematoxilina y eosina, 40X. C. Se demuestran abundantes bacilos de Hansen, aislados o formando globias. Fite-Faraco, 80X.

Se hizo el diagnóstico clínico de neurofibromatosis, el cual fue ratificado en la biopsia de una lesión de la pierna. La paciente fue remitida a Barranquilla para consulta con dermatólogos y neurólogos. Se solicitó la revisión de la biopsia, con lo cual se ratificó el diagnóstico de neurofibromatosis. Los dermatólogos solicitaron una nueva revisión del espécimen y, así, se diagnosticó la enfermedad de Hansen multibacilar con características de lepra histioide (figura 2).

**Figura 3:**
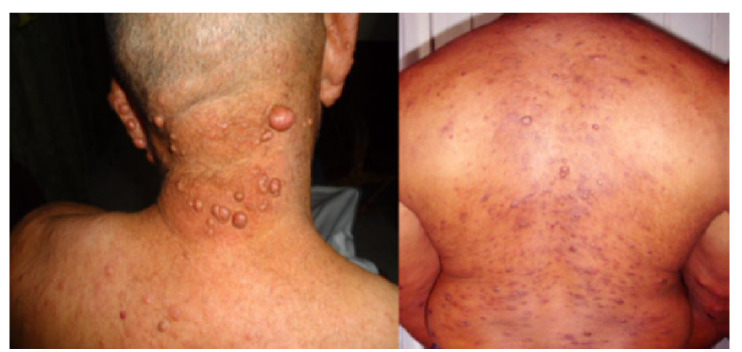
El padre de la niña presentaba múltiples pápulas y nódulos **(A),** que disminuyeron de tamaño al año de tratamiento **(B).**

*Casos 2 y 3.* A raíz del diagnóstico del caso 1, se hizo una visita familiar en la que se encontró que su padre, de 47 años, presentaba incontables nódulos de muchos años de evolución, anestésicos, cuya biopsia demostró también lepra histioide (figura 3). Su esposa y sus dos hijos varones no presentaban lesiones clínicas.

Tanto el padre como la adolescente recibieron el tratamiento recomendado por la Organización Mundial de la Salud (OMS), el cual permitió la resolución satisfactoria de las lesiones en la hija y una disminución importante del tamaño de los nódulos en el padre (figura 3B), sin que se observara persistencia bacilar en los controles.

En el 2013, una comisión médica de Sanidad Militar de las Fuerzas Armadas de Colombia visitó Providencia y constató que el padre y su hija presentaban mejoría de sus lesiones. En el examen de la madre y de los hermanos de la niña no se encontraron lesiones de lepra en ellos.

El seguimiento de la familia permitió demostrar, en el 2017, que un hijo del paciente presentaba lesiones papulares de dos años de evolución, las cuales correspondían a enfermedad de Hansen multibacilar, lepromatosa, probablemente histioide. Con este, se completaron tres casos de lepra multibacilar en la misma familia.

**Figura 4:**
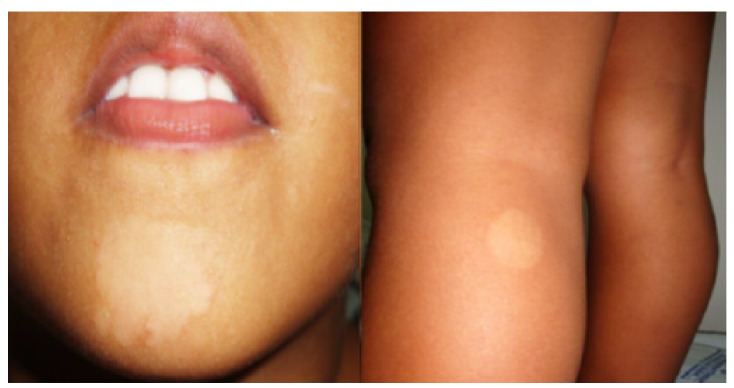
Niña de 7 años de edad con manchas hipocrómicas en el mentón y en una pierna

*Caso 4.* En la visita hecha en el 2013, se examinó a una niña de 7 años, sin contacto conocido con los tres pacientes anteriores, que presentaba una mancha hipocrómica en la cara y otra en la pierna (figura 4), con alteración dudosa de la sensibilidad, cambios que hacían sospechar la presencia de lepra indeterminada.

Se tomó una muestra de moco nasal para investigar la presencia de ADN de *Mycobacterium leprae* por reacción en cadena de la polimerasa (PCR), cuyo resultado fue positivo, y una muestra de sangre para detectar anticuerpos IgM contra el GLP-1 del bacilo de Hansen, con resultado negativo, lo cual confirmó que la niña tenía lepra indeterminada. Su localización, manejo y seguimiento han estado fuera de nuestro alcance, a pesar de los esfuerzos realizados.

Durante esa misma visita, también se tomaron muestras de moco nasal y de suero del padre y la hija con lepra histioide, así como de cuatro convivientes en contacto con los dos, incluidos la madre y los hermanos del caso índice, cuyos resultados se muestran en el cuadro 1.

**Cuadro 1:** Resultados serológicos y moleculares de los convivientes y contactos del caso índice

Número	Característica	Convivencia	PCR en muestra de moco nasal	Anti-GLP-1 en suero
1	Padre		ND	+++
2	Caso 1, hija	Sí	+	+++
3	Hijo del caso 2	Sí	-	-
4	Hijo del caso 2	Sí	-	-
5	Esposa del caso 2	Sí	+	-
6	Primo del caso 2	¿?	-	-

Anti-GLP-1: anticuerpos contra el GLP-1; ND: no determinado

## Discusión

San Andrés y Providencia son islas colombianas en el mar de las Antillas. San Andrés tiene 68.283 habitantes ^12^ y se habían han registrado casos de lepra en inmigrantes provenientes del interior colombiano. Providencia tiene 5.037 habitantes ^12^ y no se habían registrado casos de lepra allí. Los pacientes que reportamos representan una prevalencia de 8 casos por 10.000 habitantes, lo que ubica a Providencia como un sitio hiperendémico. Según la OMS, el criterio de eliminación y control de la enfermedad establece que debe haber menos de un caso por 10.000 habitantes ^13^.

Este estudio demostró la clara necesidad de impulsar actividades de búsqueda y detección de casos clínicos y de personas con infección subclínica, así como actividades de educación en salud comunitaria para impedir una mayor extensión de la enfermedad ^14^. Hasta ahora solo se han practicado algunos exámenes clínicos en unas pocas viviendas.

Según las proyecciones de población del censo del 2005, en Providencia la población menor de 15 años representa el 30 % del total ^12^ y esta constituye la población con mayor propensión a contraer la lepra ^4^^,^^5^. Por otra parte, la lepra histioide es la presentación más bacilífera entre las formas multibacilares y puede aparecer *de novo*, como en los pacientes de nuestro estudio, o corresponder a una recidiva de lepra multibacilar, con la posibilidad de ser resistente a medicamentos como la dapsona o a la rifampicina ^10^^,^^15^. Llama la atención el hecho de que, en el 2013, un hijo del paciente adulto no fue positivo en la PCR del moco nasal, ni presentó anticuerpos anti-GLP-1, pero dos años después desarrolló lepra multibacilar, que está siendo tratada adecuadamente. Este hallazgo podría indicar que, en realidad, existen más casos bacilíferos sin tratamiento en el entorno familiar o social.

El ejemplo paradigmático de lo que podría ocurrir con la lepra en una isla, es lo sucedido en Nauru, una isla de 1.200 habitantes en el Pacífico y sin historia previa de lepra, a donde llegó una paciente con lepra lepromatosa en 1912. Para el año 1920, había cuatro pacientes con lepra y, en 1925, ya eran 368. La epidemia comenzó a declinar lentamente, pero aún en 1981 el porcentaje era del 1 % ^6^^,^^7^. En este sentido, cabe resaltar que el costo social, económico y de sufrimiento humano causado por un caso no detectado ni tratado oportunamente, puede resultar mucho más elevado que implementar las actividades disponibles de búsqueda y vigilancia.

Aunque la adolescente y su padre han presentado mejoría clínica aparente de sus lesiones, persisten sus títulos de anticuerpos contra el GLP-1 y no hay datos iniciales para comparar. Esto exige que su control clínico y por laboratorio continúe, pues la persistencia de títulos altos de anticuerpos anti- GLP-1 indica permanencia de la enfermedad ^10^.

## Conclusiones

La lepra en Providencia merece mayor atención que la que ha recibido hasta ahora y sería posible abordarla en forma de estudios colaborativos multidisciplinarios que incluyan la revisión clínica, la investigación de anticuerpos anti-GLP-1, y los estudios moleculares y genéticos de la población expuesta, con miras a evaluar las posibilidades de implementar la inmunoprofilaxis o la quimioprofilaxis, según sea necesario ^16^.

## References

[1] Instituto Nacional de Salud Boletín Epidemiológico Semanal, número 52 de 2016.

[2] Rodríguez G, Pinto R (2007). La lepra. Imágenes y conceptos.

[3] Moet FJ, Pahan D, Schuring RP, Oskam L, Richardus JH (2006). Physical distance, genetic relationship, age, and leprosy classification are independent risk factors for leprosy in contact of patients with leprosy. J Infect Dis.

[4] Job CK, Jayakumar J, Kearney M, Gillis TP (2008). Transmission of leprosy: A study of skin and nasal secretions of household’s contacts of leprosy patients using PCR. Am J Trop Med Hyg.

[5] Smith WCS, Smith CM, Cree IA, Jadhaw RS, Mac Donald M, Edgard VK (2004). An approach to understanding the transmission of Mycobacterium leprae using molecular and immunologic methods: Results from de MILEP2 study. Int J Lepr Other Mycobact Dis.

[6] Richardus JH, Pahan D, Johnson RC, Smith WC, Scollard DM, Gillis TP (2016). Leprosy control. The International Texbook of Leprosy.

[7] Bray GW (1930). The story of leprosy at Nauru. Proc R Soc Med.

[8] Grant A (1934). Leprosy at Nauru since 1928. Int J Lepr Other Mycobact Dis.

[9] Guerrero MI, Arias MT, Garcés MT, León CI (2002). Desarrollo y aplicación de una prueba de RCP para detectar la infección subclínica por Mycobacterium leprae. Rev Panam Salud Pública.

[10] Penna ML, Penna GO, Iglesias PC, Natal S, Rodrigues LC (2016). Anti-PGL-1 positivity as a risk marker for the development of leprosy among contacts of leprosy cases: Systematic review and meta-analysis. PLoS Negl Trop Dis.

[11] Scollard DM (2008). The biology of nerve injury in leprosy. Lepr Rev.

[12] Departamento Administrativo Nacional de Estadística (2005). Demografía regiones Colombia.

[13] Organización Mundial de la Salud Estrategia mundial para la eliminación de la lepra 2016- 2020.

[14] Guerrero MI (2017). Vigilancia epidemiológica de lepra aplicable a sitios de baja prevalencia: una necesidad vigente. Infectio.

[15] Rodríguez G, Henríquez R, Gallo S, Panqueva C (2015). Histoid leprosy with giant lesions of fingers and toes. Biomédica.

[16] Franco-Paredes C, Rodríguez-Morales AJ (2016). Unsolved matters in leprosy: A descriptive review and call for further research. Ann Clin Microbiol Antimicrob.

